# A systematic review of clinical audit in companion animal veterinary medicine

**DOI:** 10.1186/s12917-016-0661-4

**Published:** 2016-02-26

**Authors:** Nicole Rose, Lorraine Toews, Daniel S. J. Pang

**Affiliations:** Western Veterinary Specialist and Emergency Centre, Calgary, AB Canada; Health Sciences Library, University of Calgary, Calgary, AB Canada; Veterinary Clinical and Diagnostic Sciences, Faculty of Veterinary Medicine, University of Calgary, Calgary, AB Canada; Hotchkiss Brain Institute, University of Calgary, Calgary, AB Canada

**Keywords:** Clinical audit, Systematic review, Veterinary, Audit

## Abstract

**Background:**

Clinical audit is a quality improvement process with the goal of continuously improving quality of patient care as assessed by explicit criteria. In human medicine clinical audit has become an integral and required component of the standard of care. In contrast, in veterinary medicine there appear to have been a limited number of clinical audits published, indicating that while clinical audit is recognised, its adoption in veterinary medicine is still in its infancy. A systematic review was designed to report and evaluate the veterinary literature on clinical audit in companion animal species (dog, cat, horse). A systematic search of English and French articles using Proquest Dissertations and Theses database (February 6, 2014), CAB Abstracts (March 21, 2014 and April 4, 2014), Scopus (March 21, 2014), Web of Science Citation index (March 21, 2014) and OVID Medline (March 21, 2014) was performed. Included articles were those either discussing clinical audit (such as review articles and editorials) or reporting parts of, or complete, audit cycles.

**Results:**

The majority of articles describing clinical audit were reviews. From 89 articles identified, twenty-one articles were included and available for review. Twelve articles were reviews of clinical audit in veterinary medicine, five articles included at least one veterinary clinical audit, one thesis was identified, one report was of a veterinary clinical audit website and two articles reported incomplete clinical audits. There was no indication of an increase in the number of published clinical audits since the first report in 1998. However, there was evidence of article misclassification, with studies fulfilling the criteria of clinical audit not appropriately recognised. Quality of study design and reporting of findings varied considerably, with information missing on key components, including duration of study, changes in practice implemented between audits, development of explicit criteria and appropriate statistical analyses.

**Conclusions:**

Available evidence suggests the application and reporting of clinical audit in veterinary medicine is sporadic despite the potential to improve patient care, though the true incidence of clinical audit reporting is likely to be underestimated due to incorrect indexing. Reporting standards of clinical audits are highly variable, limiting evaluation, application and repeatability of published work.

**Electronic supplementary material:**

The online version of this article (doi:10.1186/s12917-016-0661-4) contains supplementary material, which is available to authorized users.

## Background

Clinical audit is a quality improvement tool that facilitates the evaluation and improvement of care [1, 2]. In human medicine, it has become a standard of care required by some national governing bodies [3] and its implementation has yielded measurable improvements in clinical care [4–8]. Furthermore, the absence of clinical audit has been identified in allowing persistent poor performance, resulting in failures of care [9]. In veterinary medicine, national level audit with explicit criteria and reporting standards set by governing bodies does not currently exist. The only nationally mandated audit the authors are aware of is the requirement that clinics participating in the Royal College of Veterinary Surgeons Practice Standards Scheme perform audits to evaluate clinical outcomes (with the implication that these are limited to individual clinics) [10], though many veterinary clinics do perform informal audits through practices such as mortality and morbidity rounds. A formal clinical audit, first described in veterinary medicine by Mosedale [2], is centred around a structured audit cycle. In its simplest form, a clinical audit can be described by a 4 step process: Plan-Do-Study-Act (PDSA) [11–14]. This process is cyclical in nature to allow for continuous monitoring of performance and improvement [1, 11–14]. In the “Plan” step, a clear problem is identified [14]. The problem can be an outcome, such as anesthetic mortality rate, or a process associated with achieving an outcome, such as the number of patients receiving a physical examination prior to general anesthesia. When a clinical audit focuses on an outcome or process it may be described as an “outcome [clinical] audit” or “process [clinical] audit”, respectively. The “Do” step is the process of data collection [1, 11–14]. Following data collection, the “Study” step is analysis of the data followed by comparison of results to a predetermined set of standards (“explicit criteria”) [11, 14–16]. Finally, the “Act” step is the development of recommendations based upon the outcome of "Study". These recommendations are then implemented and additional audit cycles performed [1, 11–14]. In summary, clinical audit compares a current standard of care against clearly defined criteria and uses this information to drive change with the goal of improvement in patient care [1, 14]. The process should be cyclical, with regular re-evaluation to achieve the goal of continual improvement [1, 12, 14, 17].

The key principles of clinical audit are the selection of explicit criteria and its cyclical nature. Selected criteria should be easily measured, relevant to the process/outcome of interest, based on available evidence and clearly defined (“explicit”). Where there is insufficient evidence to identify criteria, clinical audit allows for performance comparison with other institutions (“benchmarking”). The tracking of performance through regular audit creates an “audit cycle”, resulting in a continuous cycle of improvement [1, 17]. Absence of a cyclical process, as is common with traditional audit (audit of clinical practice), results in a limited ability to generate continuous improvement [18]. This is an important distinction; due to the absence of explicit criteria (as defined above) and a cyclical evaluation of performance an audit of clinical practice often takes the form of a simple survey of practice and lacks the ability to drive and monitor improvement through a cyclical process.

Clinical audit has been variably defined in veterinary and human medicine [1, 11, 12, 19, 20]. Burgess [11] defines clinical audit as “a quality improvement process that seeks to improve patient care and outcomes through systematic review of care against explicit criteria and the implementation of change” [11]. Since an initial description in 1998 [2], it is unclear to what extent clinical audit is being reported in the veterinary literature and how closely reported studies adhere to the principles of an audit cycle. The purpose of this study was to perform a systematic review of the reporting of clinical audit in the veterinary literature related to companion animals (dogs, cats and horses).

## Methods

A systematic review was performed in accordance with the Preferred Reporting Items for Systematic Reviews and Meta-Analyses (PRISMA) guidelines [21]. The Cochrane Collaboration’s Cochrane Handbook of Systematic Reviews of Interventions (version 5.1.0) 2011, which is widely recognized as the gold standard for conducting systematic reviews, was used to guide our processes, along with guidance from the Centre for Reviews and Dissemination [22, 23].

Key search concepts and synonyms were developed by the review team (NR, DP). A health sciences librarian (LT) refined the search strategy by conducting iterative database queries. To ensure a high degree of search sensitivity, a combination of subject headings and text words were used and the search strategy was adapted to the search interface and indexing conventions of each database.

The following electronic databases were searched to identify journal literature and conference papers: OVID CAB Abstracts 1910 to 2014 (searched March 21, 2014 and April 4, 2014), OVID MEDLINE 1946–2014 (searched March 21, 2014), Scopus 1960 to 2014 (searched March 21, 2014) and Web of Science 1899 to 2014 (searched March 21, 2014). Search results for all databases were limited to studies published in English or French. No study design, publication type or publication date limits were applied to the search results.

As veterinary conference papers and proceedings are very selectively indexed in research databases (not all papers may be indexed for a given year, nor is indexing consistent from year to year), and relevant full-text conference proceedings are often not available (library subscriptions are prioritised to peer-reviewed journals), to identify additional unpublished studies we also searched the International Veterinary Information Service (IVIS) website tables of contents for the Annual American Association of Equine Practitioners and the British Equine Veterinary Association conference proceedings, using the terms: “audit”, “governance”, “event” and “incident”. The IVIS website did not provide access to abstracts for any of the relevant small animal conferences. The ProQuest Dissertations and Theses database was searched from 1861 to February 6^th^, 2014.

The full search strategy is documented in Table 1 and Additional file 1. Search results were downloaded into a commercial reference management software program (EndNote version X6, Thomson Reuters, New York, NY, USA) and duplicates were removed manually. Reference lists of included studies were scanned to identify additional sources.

**Table 1:** Electronic database search strategy for OVID CAB Abstracts <1910 to 2014 Week 12>

1. (critical event^a^ review^a^ or critical event^a^ report^a^ or critical event^a^ audit^a^ or critical event meeting^a^ critical inciden^a^ review^a^ or critical inciden^a^ report^a^ or critical inciden^a^ audit^a^ or critical inciden^a^ meeting^a^ significant event^a^ review^a^ or significant event^a^ report^a^ or significant event^a^ audit^a^ or significant event^a^ meeting^a^).af.
2. (chart audit or chart audits or chart auditing or clinical audit or clinical audits or clinical auditing or clinical governance or medical record^a^ audit^a^ or medical audit^a^ or outcome^a^ audit^a^ or process audit^a^ or prescription^a^ audit^a^ or medicines audit^a^ or liability audit^a^).af.
3. auditing^b^
4. 1 or 2 or 3
5. exp veterinary practice^b^
6. exp veterinary services^b^
7. exp veterinary science^b^or veterinary medicine^b^or veterinarians^b^or veterinary profession^b^
8. (veterinary or veterinarian^a^ or small animal practice^a^ or large animal practice^a^ or equine practice^a^ or bovine practice^a^ or dairy practice^a^ or beef cattle practice^a^ or mixed practice^a^).af.
9. 5 or 6 or 7 or 8
10. 4 and 9
11 limit 10 to ((english or french) and (conference or journal))

“exp” denotes that the search term was exploded; ^a^indicates that the word stem and all variant endings were retrieved. “^b^”identifies the preceding words as a subject heading indexing term. The same search strategy was conducted in all databases, adapted to the search syntax and conventions of each database (see Additional file 1)

Two authors (NR, DP) independently screened all abstracts for inclusion and any disagreement resolved through discussion to reach consensus. For the purposes of this review, clinical audit as defined by Burgess [11] (“clinical audit is a quality improvement process that seeks to improve patient care and outcomes through systematic review of care against explicit criteria and the implementation of change”) was applied as the criterion standard [11]. Inclusion criteria for the final review were: 1. Articles reporting at least two of the four components of the clinical audit cycle (data collection, review of data against defined criteria, implementing change, re-auditing) and 2. Articles that discussed the process or benefits of clinical audit in veterinary medicine. Following identification of included articles, each article was read by two authors (NR, DP) and the following information extracted to facilitate critical appraisal: aim, explicit criteria, study population, collected data, analysis performed, intervention(s) implemented.

Exclusion criteria were 1. Non-veterinary studies and, 2. Clinical audit not related to individual companion animal care.

## Results

**Fig. 1:**
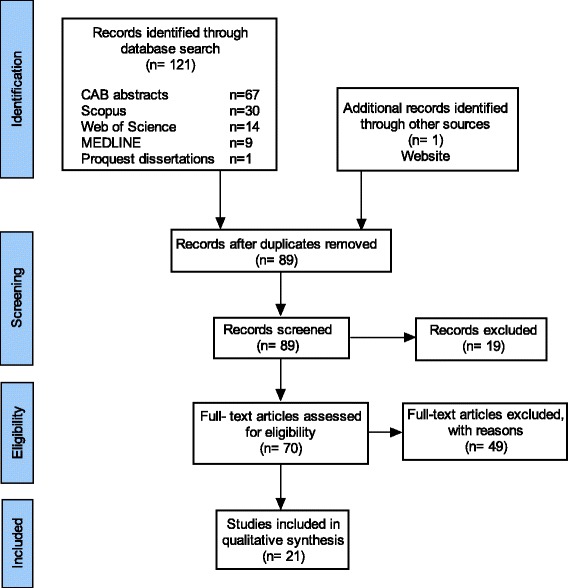
PRISMA diagram summarising literature screening process

The initial database literature research yielded 89 potential original articles (Fig. 1). Of these, 19 articles were disqualified because they were limited to the human medical literature. This resulted in 70 veterinary articles, of which 49 were excluded as they did not discuss clinical audit in companion animal care (11 food safety/biosecurity, 11 herd health, 21 business in veterinary medicine, 3 lab animal/wildlife related, 2 incidence of disease, and 1 disaster response). Initial search terms included large animal practice (Table 1 and Additional file 1) in case any clinical audits were performed where these species were treated as companion animals. With the exception of equine practice, none were identified. This resulted in 21 articles included in the analysis. The minority (*n* = 6) of these described the full audit cycle, and were therefore correctly classified as clinical audit (Table 2). These six articles described 10 separate clinical audits, the majority of which were focused on outcomes (“outcome audit”, *n* = 8) [2, 14, 20, 24, 25]. There were two clinical audits assessing the process(es) which might contribute to change (“process audit”) [20, 26]. Two articles simply described audits, without documented completion of the clinical audit cycle, such as implementation of change or re-audit [27, 28].

**Table 2:** Characteristics of articles reporting clinical audits

Reference	Audit subject	Aim	Explicit criteria	Population	Data	Data presentation & analysis	Full audit 2cycle (Y/N)	Outcome(s)/Intervention(s)	Article type
^a^Mosedale, 1998 [2]	1. Client waiting times	75 % of clients seen within 10 min of appointment time	Standard agreed within clinic	Not reported	Waiting times	Descriptive	N	1. 77.2 % on time, 83.1 % within 5 min, 93.1 % within 10 min, 13.8 % arrived late	Clinical audit – prospective design
	2. Anesthetic death	100 % survival	Published anesthetic mortality (0.14 %) rates	2282 anesthetics (species not reported)	Patient & staff id, procedure, drugs	Descriptive	N	2. 0.13 % mortality	
	3. Surgical infection	Minimal infection	Published surgical infection rates (2.5 % for clean procedures)	389 surgeries (species not reported); 241 audit 1, 148 audit 2	Dog and cat neuters, incl culture of suspected infections	Descriptive & identification of infections by surgery type/surgeon	Y	9 suspected infections (241 surgeries), with 5 (2.1 %) positive culture. Following re-training in aseptic technique, re-audit showed 0.67 % (148 surgeries) infection rate (positive culture)	
^a^Viner, 2005 [14]	Rabbit anesthesia	Reduction in anesthetic mortality rate	Not specified	158 rabbits over 3 years (year 1, 41 cases; year 2, 59 cases; year 3, 58 cases)	Anaesthetic mortality rate	Descriptive	Y	Update of anesthetic protocol – details not provided 4 % (100 cases) mortality before update, 0 % (58 cases) after, over 1 year	Clinical audit – combination of retrospective and prospective
^a^Viner, 2006 [20]	1. Diagnosis of congestive heart failure	Increased thoracic radiograph use to aid diagnosis of congestive heart failure	Expert opinion and published criteria	25 dogs	Computer records were used in all audits for an initial retrospective evaluation followed by prospective follow-up	Descriptive	Y	Radiography use increased from 84 % (*n* = 19) - 100 % (*n* = 6), through increased client communication & education	Clinical audit - Thesis (Doctorate in professional studies) Mix of retrospective and prospective study design
	2. Pruritis management	Standardised diagnosis of pruritis	Consensus discussion within clinic as well as expert opinion and published criteria. Structured approach to achieve definitive diagnosis	174 dogs	As above	Descriptive	Y	Definitive diagnosis rate increased from 56 % (*n* = 24) to 82 % (*n* = 75). Practice guidelines for establishing diagnoses established.	As above
	3. Feline hypertension screening	Improve detection of hypertension in at-risk patients	Mean arterial blood pressure (ABP) > 220 mmHg with clinical signs or mean ABP > 180 mmHg with signs (blindness, retinal haemorrhage, seizure, tachycardia)	99 cats	As above	Descriptive	Y	Use of blood pressure measurement increased from 5-83 cats over 6 months. Implemented changes unclear.	As above
	4. Renal disease management	Improve management of feline chronic renal disease	Expert opinion and published criteria. Specific diet, ACE inhibitor, regular blood work	4 cats	As above	Descriptive	N	Failed due to insufficient cases (*n* = 4)	As above
	5. Performance of obesity clinic	Improve owner compliance in attending weight loss clinic for pets and meeting targets	75 % of referred animals attend weight loss clinic and 90 % of animals achieving weight loss target	Unclear	As above	Descriptive	Y	Failed to achieve criteria (52 % attendance). Workflow to refer patients to obesity clinic described.	As above
	6. Complications following neutering	Reduce post-operative complications	Consensus discussion within clinic. Complication defined as deviation from uneventful recovery	90 dogs, 149 cats	As above	Descriptive	Y	Reduction in post-operative complications from 47-36 % (bitch spay), 8.3-1.8 % (cat spay). Increase in complications from 26-29 % (dog castration). 0 % complications (cat castration. Log books available to record audit data.	As above
^a^Viner, 2010 [24]	Use of blood pressure measurement in cats	Improve identification of hypertension in cats	Not specified – targeted at “high risk” cases (chronic nephritis, hyperthyroidism, cardiomyopathy)	79 cats over 3 x 2 month periods (month 1, 6 cases; month 2, 22 cases; month 3, 51 cases)	Number of blood pressure measurements billed	Descriptive	Y	First 2 months: 6 blood pressure measurements, 2^nd^ 6 months: 22 measurements, 3^rd^ 2 months: 51 measurements	Clinical audit – retrospective and prospective
Dunn and Dunn, 2012 [26]	Antibiotic use in small animal clinic	Appropriate prescribing of fluoroquinolone antibiotics	Published guidelines for antibiotic use: clinical evidence of infection, culture and sensitivity testing with tailored therapy	89 cases (cats and dogs; 72 cases over 12 months [audit 1], 17 cases over 3 months [audit 2])	Computer records search for antibiotic prescription	Descriptive	Y	Clinic meeting – discuss rationale for antibiotic use guidelines and adherence to policy. Re-audit showed improvement amongst permanent staff and poor performance from a locum veterinarian unaware of practice policy	Clinical audit – retrospective and prospective
Elliston et al., 2012 [27]	Post-operative wound healing in small animal clinic	Identify suspected wound infections associated with different suture materials	None described	115 dogs, 170 cats undergoing ovariohyste-rectomy	Case records	Risk ratio, Chi square and Fisher’s exact tests	N	Identified increased risk of wound infection when catgut used for subcutaneous or muscle closure (RR 3.8 and 7.6, respectively)	Audit – retrospective cohort study
Akinrinmade and Adekunle, 2012 [28]	Perioperative antibiotic use in veterinary teaching hospital.	Evaluate compliance of prophylactic use of antibiotics for surgery	General recommendations for perioperative antibiotic use	108 small animals (species not specified)	Case records – minimum data set required described	Descriptive	N	Justification of antibiotic use in only 35 % of cases. Antibiotics administered for clean procedures without justification. Rationale for antibiotic type and duration of use unclear. Many records did not meet requirement for minimum data set	Audit - restrospective
Proot and Corr, 2013 [25]	Surgical complication rate associated with learning a stifle surgical procedure	To apply quality control analysis (cumulative summation) to assess surgeon performance	Published criteria for surgical complication rates used to set limits for analysis. Criteria redefined (more stringent) as training progressed	122 dogs (167 stifle surgeries)	Consecutive records, 6 months follow up, defined of retrospective complications	Control chart (cumulative summation curve)	Y	Performance improved after 22 procedures (failure rate was acceptable during these procedures) and remained acceptable (more stringent criteria) for the remaining 145 procedures	Clinical audit - retrospective

A full clinical audit cycle is defined as an initial audit (comparing measures of interest against defined criteria), followed by data analysis and proposed intervention(s), and at least one follow-up audit to assess impact of intervention(s). Articles not fulfilling the definition of clinical audit are identified as “audit”. Interventions are changes implemented during the audit cycle with the aim of improving care. Criteria are standards against which collected data are compared. ^a^Article includes both a review and an example of clinical audit (article only included in Table 2). *NA*, Not applicable

**Fig. 2:**
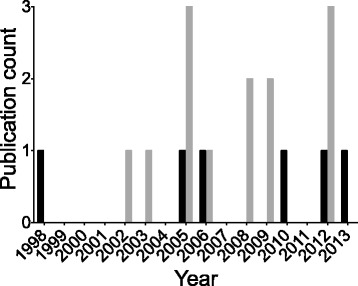
Histogram showing the sporadic publication rate of clinical audits in veterinary medicine (from studies reported in Table 2 [black bars] and 3 [grey bars]). Literature searches conducted between February – April, 2014

The majority (*n* = 13) of the identified articles were reviews of clinical audit and its application to clinical practice (Table 3). The publication dates of clinical audits and reviews are sporadic with no clear pattern of increase in recent years (Fig. 2).

**Table 3:** Characteristics of articles describing or reviewing the application of clinical audit in veterinary medicine (without reporting clinical audit data)

Reference	Summary	Article type
Rayment, 2002 [12]	Discusses clinical audit in human and veterinary medicine	Review
Moore et al., 2003 [47]	Discusses the use of clinical audit and role of continuing education in improving performance	Review
Viner et al., 2005 [17]	Describes implementation of audit in human medicine as a framework for widespread adoption in veterinary medicine	Review
SPVS/PDF MSc veterinary clinical audit group, 2005 [48]	Provides tools and education for veterinarians to perform clinical audit and contribute to national audit	Website
Mair et al., 2005 [49]	Discusses creating a colic database to help create guidelines for performing clinical audit	Editorial
Mair, 2006 [50]	Update on clinical audit in equine practice and role of evidence based medicine in determining standards	Editorial
Mair et al., 2008 [51]	Results from a survey of large animal surgeons to assess the feasibility of creating a national colic database	Survey
Godsall, 2008 [52]	Discusses the benefits of clinical governance and clinical audit in veterinary medicine	News article
Mair, 2009 [19]	Review of clinical governance and clinical audit and proposal for an international equine colic database	Review
Viner, 2009 [53]	Review of clinical audit and its role in clinical governance	Review
Dunn, 2012 [40]	Discusses clinical audit in human and veterinary medicine	Review
Viner, 2012 [54]	Discusses role of clinical governance in veterinary medicine	Book Chapter
O’Neill, 2012 [55]	Describes an electronic database (VetCompass) and a potential role in developing a national level resource for clinical audit	Review

Though the definition of clinical audit was not consistent between papers, they share the common theme of describing a key component of an audit cycle: sustained and ongoing improvement of care

### Sample size calculations

The most prevalent form of data analysis was descriptive, in the form of summary data (e.g. total number of affected cases) or percentages [2, 14, 20, 24, 26]. Where percentages were reported, none of the identified studies applied suitable statistical analyses to evaluate the reported percentage changes, such as Chi squared test or Fisher’s exact test. Analysis (Fisher’s exact test) of published data revealed that the description by Viner [24] of an increased implementation of arterial blood pressure recording was the only clinical audit to show a statistically significant improvement (*p* < 0.0001; note that an assumption was made that the sample size of the second audit equalled that of the first, as this information was not provided) [24].

In contrast, analysis of reports by Mosedale [2], Dunn and Dunn [26] and Viner [14] did not support the claimed improvements (under the following assumptions: two-tailed test with alpha and beta levels of 0.05 and 0.8, respectively) [2, 14, 26].

A documented reduction in post-operative infection rates was not statistically significant (*p* = 0.41, Fisher’s exact test); sample sizes of approximately 370 surgeries (sample sizes of 147 and 236 were collected) would be required to identify a 25 % change in infection rates [2]. The sample size during the second audit (*n* = 6) of Dunn and Dunn [26] was only sufficient to detect a change in antibiotic use of 75 %. The difference between audits had a *p* value of 0.65 (Fisher’s exact test) and a sample size of 60 would have been required to detect a change of 25 %. Analysis (Fisher’s exact test with Bonferroni’s correction) of an effort to improve peri-anaesthetic mortality rates in rabbits as a result of an unspecified change in anaesthetic protocol revealed *p* values ranging from 0.51-1.00 for pairwise comparisons between the three audits performed [14].

## Discussion

We applied the definition of Burgess [11], that “clinical audit is a quality improvement process that seeks to improve patient care and outcomes through systematic review of care against explicit criteria and the implementation of change” [11], to analyse the identified studies (Table 2). We focused specifically on study design, quality of reporting, evidence of a cyclical process and implementation of change, definition and application of explicit criteria, and systematic review of care including the use of appropriate statistical testing.

Overall, the majority of identified audits suffered from weaknesses in study design and reporting, with resultant limitations on the possibility of identifying real improvement and the repeatability of audits.

### Study design

The most common study design employed was a retrospective audit of clinical practice followed by an intervention and prospective audit (Table 2) [14, 20, 24, 26]. The reliance on retrospective data collection is limited by a dependence on record quality; the accuracy and completeness of records specific to the clinical audit subject as well as essential supporting information such as the population of animals presented to a clinic (a necessary denominator for study assessment). Many of the clinical audits identified relied on searches of computerised records to identify cases for audit. This has the considerable advantage of speed but the disadvantage of relying on accuracy in entering the appropriate key word(s) associated with the audit subject. It is likely that accuracy of tagging case records in a clinic and the type of computerised billing or database software employed [20]. Additionally, a key word search could lead to a single animal/case being counted numerous times if the procedure being studied was performed multiple times in the same animal [20, 24]. One approach employed to deal with this issue was to focus the clinical audit on the performance of a procedure, such as number of arterial blood pressure measurements performed, rather than the number of unique cases handled [20, 24]. When working with computerised records, a prospective clinical audit design provides the potential for educating staff in the importance of consistent and accurate records, allowing a more accurate comparison of different audit periods. Where computerised search (retrospective) for the initial audit preceded a prospective audit, it is difficult to identify inaccuracies in computerised data recording without some comparison of case numbers [20]. This problem is compounded when the subject of the clinical audit occurs uncommonly during the study period e.g. chronic kidney disease in cats [20].

Several of the identified studies reported a prospective study design, commonly using a simple handwritten log, with the advantage of focused data collection [2, 20, 25]. Ideally, though often limited by labour costs, data collection would be performed by an independent observer to avoid selective reporting. In prospective studies, a possible confound of participants knowing they are being observed should be considered, as being under observation may change behaviour in a way that influences outcome (Hawthorne effect) [29]. The occurrence and impact of behavioural changes will be easier to track if the clinical audit focuses on the process(es) of care (“process audit”) rather than an outcome measure (“outcome audit”). Where retrospective and prospective approaches are included within the same clinical audit [14, 20, 24, 26], unless processes are clearly described in both retrospective and prospective arms, it is difficult to evaluate the impact of the Hawthorne effect and interpret the results.

The majority of identified clinical audits were outcome-based. While it is tempting to focus on outcome (e.g. return of spontaneous circulation following cardiac arrest), as it is the ultimate measure of failure/success, unless the steps (processes) of clinical care are described and recorded (e.g. frequency of chest compressions) it is impossible to compare clinical audits, replicate studies and maximise learning [20, 25]. Furthermore, where the key process(es) associated with a successful outcome are unknown, limiting focus to outcomes limits evaluation of the clinical audit when an improvement in outcome is not achieved. Conversely, when an outcome audit achieves improvement, unclear documentation of processes inhibits identification of potentially contributing processes [14]. Though clinical audits are not designed to establish a causal relationship, accurate reporting will help identify areas for further improvement and research.

### Quality of reporting

In general, reporting quality was highly variable between studies with deviations from expected standards in scientific reports. These limitations make it difficult, if not impossible, to evaluate results, compare findings between clinical audits or replicate clinical audits. Reporting deficits ranged from not identifying the species studied, not describing the criteria/methods of case selection (including omission of the number of eligible cases versus those collected), neglecting a detailed description of the changes implemented during the clinical audit cycle or intervening time between audits [2, 14, 20, 24–26]. Those audits that formed the focus of a paper, rather than being included in a general review, tended to follow a higher standard of reporting [25, 26].

As a result of the limitations described above, as well the limited power of many of the identified audits (see Systematic review of care, below), it is difficult to evaluate the claimed improvements in care. Where failure to achieve improvements were identified common causes included poor owner compliance, selection of an uncommon disease, and limited statistical power [20].

### Cyclical process and implementation of change

Of the 15 audits reported in 8 publications, five audits (33 %) failed to demonstrate that a cyclical process had been followed [2, 20, 27, 28]. Furthermore, in 2 (20 %) of the audits that included a cyclical process it was unclear which interventions or changes were implemented between audit cycles [14, 20]. Claimed improvements were made by four of the published audits, though further analysis revealed that only one may have had sufficient power [24]. In contrast to a patient-centred approach, Proot and Corr [25] focused on documenting an improvement in individual performance, using a statistical process control chart approach to track performance (see Systematic review of care, below) [25].

### Explicit criteria

The role of identifying explicit criteria as a standard of care allows comparison of performance [1].

Sources of explicit criteria vary depending on the availability of high quality evidence or peer-reviewed consensus guidelines. The identified studies report 3 broad types of criteria: those resulting from consensus discussion within a clinic [2], those based on published guidelines or evidence [2, 25, 26] and criteria based on a combination of these latter approaches [20]. Where explicit criteria were not applied or reported [14, 24, 27, 28], improvements in performance can still be demonstrated but may be misleading if they are below published standards. Conversely, performance exceeding published standards that goes unrecognised limits the opportunity to learn from, or apply, successful practice. Ideally, explicit criteria should be based on available high quality evidence. In the face of the limited evidence available in many areas of veterinary medicine, consensus guidelines combining available evidence with expert interpretation produced by professional bodies can be used [30–33]. In contrast, guidelines developed within a single clinic are likely to be limited by the smaller pool of experience and expertise available.

### Systematic review of care

Review of care was limited by over-interpretation of percentage changes without appropriate statistical analyses. In particular, the reporting of percentages in the presence of small sample sizes is misleading as a small change in numbers will have a dramatic effect on the resultant percentage [20, 24, 26]. Subsequent analyses revealed that only one of the identified clinical audits resulted in a statistically significant change [24].

Additionally, while it is tempting to select an easily measurable and emotionally-charged outcome, such as anaesthetic death, as the subject of a clinical audit, where such events are rare it is difficult to achieve an appropriate sample size within a single centre.

Despite the sample size limitations some audits were successful in identifying several potential areas of improvement within each clinic: Mosedale [2] and Dunn and Dunn [26] both recognised the potential for individual performance to have a negative impact on clinical practice through poor aseptic technique, adherence to clinical guidelines and communication (Table 2) [2, 14].

Viner’s [20] doctoral thesis clearly reported the cyclical clinical audit process and iterative approach which was often used to achieve improvements in performance [20]. This body of work is particularly instructive in highlighting factors contributing to unsuccessful clinical audits, including client communication and compliance (clinical audit of a weight loss clinic), case identification and recruitment (clinical audit of feline chronic renal disease management), loosely defined criteria of care (clinical audit of post-operative complications). These failures, as with negative study results in biomedical research, are seldom reported in the literature but have a role to play in learning the pitfalls of conducting clinical audit [34].

One study applied a statistical process control chart to evaluate performance [25]. Such an approach is relatively novel in human and veterinary medicine and originates from quality control procedures for production systems [35]. This, and similar statistical techniques, allows very rapid feedback on performance and can be applied to document the learning curve associated with a new technique, the consistent performance of a skill and as a means to facilitate early identification of poor performance as a result of dramatic failures in care [36, 37].

### Failure and success of clinical audits

There were two instances of clinical audit failure recorded in the studies identified; management of chronic kidney disease in cats and performance of a weight loss clinic [20]. These highlighted two different pitfalls: selection of a feasible subject and achieving change in complex systems. In the former, subject selection can be guided by either a pilot study or examination of retrospective data to identify feasibility [1, 11, 38]. When the selected subject is part of a complex system, the risk of failure can be minimised by identifying the processes associated with the outcome of interest [1, 11]. Additionally, an early realisation that changes in human behaviour may be necessary, though hard to achieve, can help design a feasible study. Behavioural barriers, staff communication and record keeping were identified by Viner during evaluation of the clinical audit of the weight loss clinic [20].

Rayment [12] identified the following benefits to implementing clinical audit in veterinary medicine: a reduction of clinical error, the promotion of high standards of care, a gain in the confidence of clients and the promotion of team building amongst staff members [12]. Many of these benefits were described in the identified audits [2, 14, 20, 24, 26]. From these, several important lessons can be learned.

Objective data collection was essential in both identifying sub-standard care and assessing the effect on outcomes when changes were implemented [2, 14, 20, 24, 26]. Without data collection identification of deficits requires marked deviation from normal practice. Such deviation may be difficult to identify when a clinic is assessed as a whole, where the performance of individuals within the group may not be obvious [2, 26]. Furthermore, clinical audit introduces objectivity in recording data, eliminating the inherent risks of relying on memory and confirmation bias [2]. The collected data can form the basis for discussion at staff meetings and allow for input from various parties regarding how care can be improved [26]. Successful clinical audit is predicated on a supportive environment and staff involvement at all levels [39]. This is reflected by the association between a multidisciplinary approach (not limited to veterinarians) and improved practice [2, 14, 20, 24, 40]. Where staff changes occur, audit can confirm that acceptable standards are being maintained, avoiding the assumption that clinic policies will be followed [26]. Where current practice meets acceptable standards, clinical audit can serve as positive reinforcement at a team or individual level [17, 26].

Maintaining the audit cycle over the long term can be difficult. Of the identified studies, a minority were performed for longer than a few months [14, 20, 25]. However, as evident from large-scale audits in production animals, with appropriate support sustained improvement is achievable [41, 42].

### Future of clinical audit

Though clinical audit is clearly recognized and implemented in veterinary medicine, the small number of published studies limits firm conclusions regarding the adoption and application of clinical audit from being drawn (Fig. 2). Additionally, the publication dates of identified articles (Fig. 2), do not show a clear pattern of increasing clinical audit publications. However, these data should be evaluated cautiously as it is not possible to know to what extent clinical audit occurs without dissemination in the form of an accessible publication, and in performing this systematic review it was apparent that articles fulfilling the criteria of clinical audit were not identified by the search strategies employed. These potential omissions reflect the well-recognised limitations of article indexing, affecting medicine and veterinary medicine, as well as the poor recognition of clinical audit in veterinary medicine [43]. Accurate article indexing is critical to identification of relevant articles through a structured search strategy. Indexing is based on words included in the title, abstract and key words of an article, and it is likely that many authors are not fully aware of the implications of omitting certain terms (such as “clinical audit”) on the visibility of their article. Related to this, the misuse of the term “clinical audit” adds to confusion regarding its definition [27].

Several recent examples highlight the issue of indexing [44–46]. Hofmeister et al. [46] performed a study fulfilling the criteria of clinical audit [46]. In a prospective, observational pre-post intervention study design they performed an initial audit of anesthesia patient safety incidents measured against explicit criteria (from available literature), implemented changes (related to misinjection, performance of endotracheal intubation and anesthesia machine check), and re-audited, with the outcome of creating a successful decrease in patient safety incidents (from 3.6 % to 1.4 % over the course of over 4000 anesthetic procedures, *p* < 0.0001). Furthermore, this study exemplified several of the supporting measures discussed above e.g. involvement of several levels of clinic staff (veterinarians and AHTs), prospective data collection and changes in human behavior. This study clearly fulfills the definition of clinical audit, though it was not identified through our search strategy. Broadening the search sufficiently to identify such articles would have prevented a systematic review of the literature as the resultant search would be overwhelmingly broad and unstructured, without guaranteeing that results were complete or reproducible, the cornerstone of systematic review. The solution to this problem is the correct and accurate indexing of articles through education of authors, reviewers and editors [42].

## Conclusion

Clinical audits have the potential to generate a measurable improvement in clinical practice and add to evidence-based veterinary medicine. Healthcare, in both human and veterinary medicine, is constantly evolving, indicating an increasing role for clinical audits in the future. By performing audit cycles, the quality of care offered can be monitored, evaluated and continually improved [12]. The number of clearly identifiable clinical audits in the scientific literature remains small, though the number of publications identified is an underestimation due to inaccurate article indexing.

Unfortunately, the majority of published audits failed to generate an improvement in quality as a result of weak study design. Frequent departures from recognised reporting standards limited opportunities to learn from the work performed.

## Additional file

Additional file 1:
**Database search strategies.** (DOCX 88 kb)

## References

[1] Langley G, Moen R, Nolan K, Nolan T, Norman C, Provost L. The improvement guide: a practical approach to enhancing organizational performance, 2nd edn. San Francisco, CA, USA: Jossey-Bass; 2009

[2] Mosedale P (1998). Introducing clinical audit to veterinary practice. Practice.

[3] Good medical practice [http://www.gmc-uk.org/guidance/good_medical_practice.asp]. Accessed 15 Feb 2015.

[4] Patel NK, Sarraf KM, Joseph S, Lee C, Middleton FR (2013). Implementing the National Hip Fracture Database: An audit of care. Injury.

[5] Audit NP (2008). Impact of NICE guidance on rates of haemorrhage after tonsillectomy: an evaluation of guidance issued during an ongoing national tonsillectomy audit. Qual Saf Health Care.

[6] Haynes AB, Weiser TG, Berry WR, Lipsitz SR, Breizat AH, Dellinger EP, et al. Changes in safety attitude and relationship to decreased postoperative morbidity and mortality following implementation of a checklist-based surgical safety intervention. BMJ Qual Safety. 2011;20(1):102–7. 10.1136/bmjqs.2009.040022 21228082

[7] Pronovost P, Needham D, Berenholtz S, Sinopoli D, Chu H, Cosgrove S, et al. An intervention to decrease catheter-related bloodstream infections in the ICU. New Engl J Med. 2006;355(26):2725–32. 10.1056/NEJMoa061115 17192537

[8] Shonfeld A, Riyat A, Kotecha A, Sacks M (2011). Critical care transfers: using audit to make a difference. Anaesthesia.

[9] The Stationary Office. Learning from Bristol: the report of the public inquiry into children’s heart surgery at the Bristol Royal Infirmary 1984–1995. London: The Stationary Office; 2001.

[10] Royal College of Veterinary Surgeons. Practice standards scheme manual. London: Royal College of Veterinary Surgeons; 2014. [http://www.rcvs.org.uk/document-library/pss-review-2015-small-animal-draft-modules/]. Accessed 8 Jan 2014.

[11] Burgess R (2011). New Principles of Best Practice in Clinical Audit.

[12] Rayment K (2002). Clinical audit - A means of evaluating ‘quality’. Practice.

[13] The Royal College of Anaesthetists. Raising the Standard: a compendium of audit recipes for continous quality improvement in anaesthesia. London: The Royal College of Anaesthetists; 2012.

[14] Viner B (2005). Clinical audit in veterinary practice - the story so far. Practice.

[15] Weissman N, Allison J, Kiefe C, Farmer R, Weaver M, Williams O, et al. Achievable benchmarks of care: the ABCs of benchmarking. J Eval Clin Pract. 1999;5(3):269–81. 10.1046/j.1365-2753.1999.00203.x 10461579

[16] Hall R, Khan F, Bayley M, Asllani E, Lindsay P, Hill M, et al. Benchmarks for acute stroke care delivery. Int J Qual Heath C. 2013;25(6):710–8. 10.1093/intqhc/mzt069 PMC3842126 24141011

[17] Viner B, Jenner C (2005). Clinical audit - learning from the medical profession. Vet Rec.

[18] Farrell C, Hill D (2012). Time for change: traditional audit or continuous improvement?. Anaesthesia.

[19] Mair TS (2009). Clinical governance, clinical audit, and the potential value of a database of equine colic surgery (Special Issue: New perspectives in equine colic). Vet Clin N Am-Equine.

[20] Viner B. Introducing clinical audit into veterinary practice. *Msc(VetGP).* London, UK: Middlesex University; 2006.

[21] Liberati A, Altman DG, Tetzlaff J, Mulrow C, Gotzsche PC, Ioannidis JP, et al. The PRISMA statement for reporting systematic reviews and meta-analyses of studies that evaluate health care interventions: explanation and elaboration. PLoS Med. 2009;6(7), e1000100. 10.1371/journal.pmed.1000100 PMC2707010 19621070

[22] Centre for Reviews and Dissemination [http://www.york.ac.uk/inst/crd/pdf/Systematic_Reviews.pdf]. Accessed 5 July 2014.

[23] Cochrane Handbook for Systematic Reviews of Interventions Version 5.1.0 [http://handbook.cochrane.org/]. Accessed 15 Feb 2015.

[24] Viner B (2010). Clinical effectiveness. What does it mean for practitioners - and cats?. J Feline Med Surg.

[25] Proot JL, Corr SA (2013). Clinical audit for the tibial tuberosity advancement procedure: establishing the learning curve and monitoring ongoing performance for the tibial tuberosity advancement procedure using the cumulative summation technique. Vet Comp Orthopaed.

[26] Dunn F, Dunn J (2012). Clinical audit: application in small animal practice. In Practice.

[27] Elliston R, Heayns B, Fish C. A clinical audit to identify factors contributing to surgical wound healing complications. Veterinary Nurse. 2012;3(3):188-95.

[28] Akinrinmade JF, Adekunle OM (2012). An audit of surgical antibiotic prophylaxis at the Veterinary Teaching Hospital, Ibadan. Niger Vet J.

[29] Parsons HM (1974). What happened at Hawthorne?. Science.

[30] Harold D, Jensen T, Johnson A, Knowles P, Meyer R, Rucinsky R, et al. 2013 AAHA/AAFP fluid therapy guidelines for dogs and cats. J Am Anim Hosp Assoc. 2013;49(3):149–59. 10.5326/JAAHA-MS-5868 23645543

[31] Behrend E, Kooistra H, Nelson R, Reusch C, Scott-Moncrief J (2013). Diagnosis of spontaneous canine hyperadrenocorticism: 2012 ACVIM Consensus Statement (Small Animal). J Vet Intern Med.

[32] Brainard B, Boller M, Fletcher D (2012). RECOVER evidence and knowledge gap analysis on veterinary CPR. Part 5: monitoring. J Vet Emerg Crit Car.

[33] Hearnshaw H, Harker R, Cheater F, Baker R, Grimshaw G (2001). Expert consensus on the desirable characteristics of review criteria for the improvement of healthcare quality. Qual Health Care.

[34] Dwan K, Altman D, Arnaiz J, Bloom J, Chan A-W, Cronin E, et al. Systematic review of the empirical evidence of study publication bias and outcome reporting bias. Plos One. 2008;3(8):1–31. 10.1371/journal.pone.0003081 PMC2518111 18769481

[35] Biau D, Williams S, Schlup M, Nizard R, Porcher R (2008). Quantitative and individualized assessment of the learning curve using LC-CUSUM. Brit J Surg.

[36] Campbell R, Hecker K, Biau D, Pang D. Student attainment of proficiency in a clinical skill: the asssessment of individual learning curves. PLoS One. 2014;9(2). 10.1371/journal.pone.0088526 PMC3930528 24586337

[37] Spiegelhalter D, Grigg O, Kinsman R, Treasure T (2003). Risk-adjusted sequential probability ratio tests: applications to Bristol, Shipman and adult cardiac surgery. Int J Qual Health C.

[38] Dixon N (1996). Good practice in clinical audit.

[39] Johnston G, Crombie IK, Davies HT, Alder EM, Millard A (2000). Reviewing audit: barriers and facilitating factors for effective clinical audit. Qual Health Care.

[40] Dunn J (2012). Clinical audit: A tool in the defence of clinical standards. Practice.

[41] Grandin T (2000). Effect of animal welfare audits of slaughter plants by a major fast food company on cattle handling and stunning practices. J Am Vet Med Assoc.

[42] Grandin T (2005). Maintenance of good animal welfare standards in beef slaughter plants by use of auditing programs. J Am Vet Med A.

[43] Dickersin K, Scherer R, Lefebvre C (1994). Systematic Reviews: Identifying relevant studies for systematic reviews. Brit Med J.

[44] Bille C, Auvigne V, Bomassi E, Durieux P, Libermann S, Rattez E (2014). An evidence-based medicine approach to small animal anaesthetic mortality in a referral practice: the influence of initiating three recommendations on subsequent anaesthetic deaths. Vet Anesth Analg.

[45] Bille C, Auvigne V, Libermann S, Bomassi E, Durieux P, Rattez E (2012). Risk of anaesthetic mortality in dogs and cats: an observational cohort study of 3546 cases. Vet Anesth Analg.

[46] Hofmeister E, Quandt J, Braun C, Shepard M (2014). Development, implementation and impact of simple patient safety interventions in a university teaching hospital. Vet Anesth Analg.

[47] Moore DA, Klingborg DJ (2003). Using clinical audits to identify practitioner learning needs. J Vet Med Educ.

[48] Clinical Audit Group Strategy [http://www.vetaudit.co.uk/]. Accessed 10 April 2015.

[49] Mair TS, White NA (2005). Improving quality of care in colic surgery: Time for international audit?. Equine Vet J.

[50] Mair TS (2006). Evidence-based medicine and clinical audit: what progress in equine practice?. Equine Vet Educ.

[51] Mair TS, White NA (2008). The creation of an international audit and database of equine colic surgery: survey of attitudes of surgeons (Special issue: colic). Equine Vet J.

[52] Godsall S (2008). Using clinical audits as tools for positive change in practice. Vet Times.

[53] Viner B (2009). Using audit to improve clinical effectiveness. Practice.

[54] Viner B, Clarke C, Chapman M (2012). Clinical governance. BSAVA Manual of Small Animal Practice Management and Development.

[55] O’Neill D (2012). VetCompass clinical data points the way forward. Vet Ir J.

