# Supplementary material for: A systematic review of clinical audit in companion animal veterinary medicine
Source: BMC Vet Res. 2016 Feb 26;12:40. doi: 10.1186/s12917-016-0661-4 (PMC4769507; doi:10.1186/s12917-016-0661-4)
Supplement: Additional file 1: — Database search strategies. (DOCX 88 kb) [file 12917_2016_661_MOESM1_ESM.docx]

**Additional file 1**: Electronic database search strategies for OVID CAB Abstracts, OVID Medline, Web of Science, Scopus and ProQuest Dissertations and Theses databases.

|  | Table 1: Electronic database search strategy for OVID CAB Abstracts <1910 to 2014 Week 13> |
| --- | --- |
| 1 | (critical event* review* OR critical event* report* OR critical event* audit* OR critical event meeting* OR critical inciden* review* OR critical inciden* report* OR critical inciden* audit* OR critical inciden* meeting* OR significant event* review* OR significant event* report* OR significant event* audit* OR significant event* meeting*).af. |
| 2 | (chart audit OR chart audits OR chart auditing OR clinical audit OR clinical audits OR clinical auditing OR clinical governance OR medical record* audit* OR medical audit* OR outcome* audit* OR process audit* OR prescription* audit* OR medicines audit* OR liability audit*).af. |
| 3 | auditing/ |
| 4 | 1 OR 2 OR 3 |
| 5 | exp veterinary practice/ |
| 6 | exp veterinary services/ |
| 7 | exp veterinary science/ or veterinary medicine/ or veterinarians/ or veterinary profession/ |
| 8 | (veterinary OR veterinarian* OR small animal practice* OR large animal practice* OR equine practice* OR bovine practice* OR dairy practice* OR beef cattle practice* OR mixed practice*).af. |
| 9 | 5 OR 6 OR 7 OR 8 |
| 10 | 4 and 9 |

The forward slash / at the end of a term indicates it is a subject heading

|  | Electronic database search strategy for **OVID Medline** <1946 to 2014 Week 11 > |
| --- | --- |
| 1 | exp Clinical Audit/ |
| 2 | Clinical Governance/ |
| 3 | (critical event* review* OR critical event* report* OR critical event* audit* OR critical event meeting* OR critical inciden* review* OR critical inciden* report* OR critical inciden* audit* OR critical inciden* meeting* OR significant event* review* OR significant event* report* OR significant event* audit* OR significant event* meeting*).af. |
| 4 | (chart audit OR chart audits OR chart auditing OR clinical audit OR clinical audits OR clinical auditing OR clinical governance OR medical record* audit* OR medical audit* OR outcome* audit* OR process audit* OR prescription* audit* OR medicines audit* OR liability audit*).af. |
| 5 | (1 or 2 or 3 or 4) not auditory.af. |
| 6 | exp Veterinary Medicine/ |
| 7 | Veterinarians/ |
| 8 | (veterinary OR veterinarian* OR small animal practice* OR large animal practice* OR equine practice* OR bovine practice* OR dairy practice* OR beef cattle practice* OR mixed practice*).af. |
| 9 | 6 or 7 or 8 |
| 10 | 5 and 9 |

The forward slash / at the end of a term indicates it is a subject heading

|  | Electronic database search strategy for **Web of Science**  *Indexes=SCI-EXPANDED, SSCI, CPCI-S, CPCI-SSH Timespan=1900-2014* |
| --- | --- |
| #1 | TOPIC: (“critical event* review*” OR “critical event* report*” OR “critical event* audit*” OR “critical event* meeting*” OR “critical inciden* review*” OR “critical inciden* report*” OR “critical inciden* audit*” OR “critical inciden* meeting*” OR “significant event* review*” OR “significant event* report*” OR “significant event* audit*” OR “significant event* meeting*”) |
| #2 | TOPIC: (“chart audit” OR “chart audits” OR “chart auditing” OR “clinical audit” OR “clinical audits” OR “clinical auditing” OR “clinical governance” OR “medical record* audit*” OR “medical audit*” OR “outcome* audit*” OR “process audit*” OR “prescription* audit*” OR “medicines audit*” OR “liability audit*”) |
| #3 | #2 OR #1 |
| #4 | TOPIC: (veterinary OR veterinarian* OR “small animal practice*” OR “large animal practice*” OR “equine practice*” OR “mixed practice*” OR “bovine practice*” OR “dairy practice*” OR “beef cattle practice*”) |
| #5 | #3 AND #4 |

|  | Electronic database search strategy for **Scopus** |
| --- | --- |
| 1 | TITLE-ABS-KEY((critical PRE/2 event* PRE/2 review*) OR (critical PRE/2 event* PRE/2 report*) OR (critical PRE/2 event* PRE/2 audit*) OR (critical PRE/2 event PRE/2 meeting*) OR (critical PRE/2 inciden* PRE/2 review*) OR (critical PRE/2 inciden* PRE/2 report*) OR (critical PRE/2 inciden* PRE/2 audit*) OR (critical PRE/2 inciden* PRE/2 meeting*) OR (significant PRE/2 event* PRE/2 review*) OR (significant PRE/2 event* PRE/2 report*) OR (significant PRE/2 event* PRE/2 audit*) OR (significant PRE/2 event* PRE/2 meeting*)) |
| 2 | TITLE-ABS-KEY((chart PRE/2 audit) OR (chart PRE/2 audits) OR (chart PRE/2 auditing) OR (clinical PRE/2 audit) OR (clinical PRE/2 audits) OR (clinical PRE/2 auditing) OR (clinical PRE/2 governance) OR (medical PRE/2 record* audit*) OR (medical PRE/2 audit*) OR (outcome* PRE/2 audit*) OR (process PRE/2 audit*) OR (prescription* PRE/2 audit*) OR (medicines PRE/2 audit*) OR (liability PRE/2 audit*)) |
| 3 | 2 OR 1 |
| 4 | TITLE-ABS-KEY(veterinary OR veterinarian* OR (small PRE/1 animal PRE/1 practice*) OR (large PRE/1 animal PRE/1 practice*) OR (equine PRE/1 practice*) OR (bovine PRE/1 practice*) OR (dairy PRE/1 practice*) OR (beef PRE/1 cattle PRE/1 practice*) OR (mixed PRE/2 practice*)) |
| 5 | 3 AND 4 |

PRE/n denotes "precedes by", where the first term in the search must precede the second by a specified number of terms (n).

|  | Electronic database search strategy for **ProQuest Dissertations and Theses** |
| --- | --- |
| 1 | (all((critical PRE/2 event* PRE/2 review*) OR (critical PRE/2 event* PRE/2 report*) OR (critical PRE/2 event* PRE/2 audit*) OR (critical PRE/2 event PRE/2 meeting*) OR (critical PRE/2 inciden* PRE/2 review*) OR (critical PRE/2 inciden* PRE/2 report*) OR (critical PRE/2 inciden* PRE/2 audit*) OR (critical PRE/2 inciden* PRE/2 meeting*) OR (significant PRE/2 event* PRE/2 review*) OR (significant PRE/2 event* PRE/2 report*) OR (significant PRE/2 event* PRE/2 audit*) OR (significant PRE/2 event* PRE/2 meeting*)) |
| 2 | (all((chart PRE/2 audit) OR (chart PRE/2 audits) OR (chart PRE/2 auditing) OR (clinical PRE/2 audit) OR (clinical PRE/2 audits) OR (clinical PRE/2 auditing) OR (clinical PRE/2 governance) OR (medical PRE/2 record* audit*) OR (medical PRE/2 audit*) OR (outcome* PRE/2 audit*) OR (process PRE/2 audit*) OR (prescription* PRE/2 audit*) OR (medicines PRE/2 audit*) OR (liability PRE/2 audit*)) |
| 3 | 2 OR 1 |
| 4 | (all(veterinary OR veterinarian* OR (small PRE/1 animal PRE/1 practice*) OR (large PRE/1 animal PRE/1 practice*) OR (equine PRE/1 practice*) OR (bovine PRE/1 practice*) OR (dairy PRE/1 practice*) OR (beef PRE/1 cattle PRE/1 practice*) OR (mixed PRE/2 practice*)) |
| 5 | 3 AND 4 |

PRE/n denotes "precedes by", where the first term in the search must precede the second by a specified number of terms (n).
